# P-920. Impact of Positive Blood Culture Antimicrobial Stewardship on Staphylococcus aureus Bloodstream Infection Management across Critical Access, Small, and Medium Community Hospitals

**DOI:** 10.1093/ofid/ofaf695.1126

**Published:** 2026-01-11

**Authors:** Ashley L Cubillos, Kellie Fortier, Andie Hoang

**Affiliations:** Banner North Colorado Medical Center, Loveland, CO; Banner Health, Phoenix, AZ; Banner North Colorado Medical Center, Loveland, CO

## Abstract

**Background:**

Evidence links specific clinical management actions with improved clinical outcomes in *Staphylococcus aureus* bloodstream infections (SABSI), making this condition a key target for antimicrobial stewardship initiatives. This study seeks to quantify the impact of an intervention focused on prospective audit and feedback of *S. aureus-*positive blood cultures on adherence to evidence-based management practices.

**Methods:**

This retrospective, pre-post analysis included patients with SABSI across 5 community hospitals, from before and after implementation of thrice-weekly prospective antimicrobial stewardship audit and feedback of positive blood cultures. The primary outcome was a composite percent completion of four treatment elements for SABSI, including serial blood cultures until documented clearance, echocardiography, targeted antimicrobial therapy within 24 hours of SABSI identification, and infectious diseases consultation. Other outcomes included appropriateness of discharge therapy agent and duration, and overall clinical outcomes, including all-cause readmission, mortality, and recurrent SABSI at 90 days.

**Results:**

Fifty patients were included in each of the pre- and post-implementation groups. The percent completion of the treatment elements numerically increased from 28/50 (56%) in the pre group, to 38/50 (76%) of the post group, though this increase was not statistically significant (p=0.057). This change was driven largely by increase in targeted treatment within 24 hours (30/50 [60%] vs. 40/50 [80%], P = 0.049). Appropriateness of discharge regimen was numerically but not significantly increased (33/41 [80%] vs. 38/42 [90%], P=0.276). No differences were noted in readmission or recurrent SABSI, however 90-day mortality was lower in the post group (6/43 [14%] vs. 1/47 [2%], P =0.044).

**Conclusion:**

Implementation of positive blood culture review in SABSI across 5 community hospitals numerically increased compliance with evidence-based treatment elements in a small retrospective pre-post cohort analysis. Appropriate management of SABSI is a key target for antimicrobial stewardship programs across hospitals of all sizes and settings.

**Disclosures:**

All Authors: No reported disclosures

